# Effectiveness of sequential lines of biologic and targeted small molecule drugs in psoriasis: A systematic review and meta‐analysis

**DOI:** 10.1002/ski2.350

**Published:** 2024-02-29

**Authors:** Charlotte E. Gollins, Rosie Vincent, Caoimhe Fahy, Neil McHugh, William Tillett

**Affiliations:** ^1^ Dermatology Department Royal United Hospitals Bath NHS Foundation Trust Bath UK; ^2^ Department of Life Sciences Centre for Therapeutic Innovation University of Bath Bath UK; ^3^ University Hospitals Bristol and Weston NHS Foundation Trust Bristol UK; ^4^ Royal National Hospital for Rheumatic Diseases Bath UK

## Abstract

To assess current evidence of effectiveness of sequential lines of biologic and targeted small molecule drugs for psoriasis beyond first line. A systematic search of the literature (Medline, Embase and bibliographic) was undertaken in October and December 2022 to find all studies assessing effectiveness of biologics and targeted small molecules when used beyond first‐line in adults with psoriasis (PROSPERO CRD42022365298). Data extraction and a bias assessment (Risk Of Bias In Non‐randomized Studies—of Interventions/Cochrane RoB2) were undertaken for all included studies. A random effects proportional meta‐analysis was undertaken for PASI75/90/100 at 12–16 weeks for each line of treatment (1st to 4th). Of 2666 abstracts identified, a full text review was undertaken of 177 studies; 20 manuscripts met eligibility criteria. Twenty studies were included in the analysis: 19 observational studies and one sub analysis of a RCT; *n* = 6495 (average age 49.7 years, female 35.1%). Eleven studies assessed second line biologic, nine assessed third + line. A meta‐analysis of PASI75 at 12–16 weeks found pooled effect percentage achieving PASI75 of 61%, 56%, 79% and 61% in 1st, 2nd, 3rd and 4th line biologics respectively. Meta‐analyses of PASI90/100 also found no evidence of diminished effectiveness with sequential lines (PASI90 46.1%, 39.9%, 55.8% and 33.7% and PASI100 36.7%, 30.3%, 46.7% and 30.4% in 1st to 4th line respectively). Available evidence for effectiveness of biologics beyond first line in psoriasis is predominantly observational, at high risk of bias and of low quality. There is very limited data for effectiveness beyond second line. Evidence indicates that biologics can be effective to fourth‐line.

## Graphical abstract

We assessed and synthesised the currently available data on response to biologic and targeted small molecule treatments in psoriasis, when used beyond first line. There is very little available data beyond second line, and data is of low quality and at high risk of bias. Available evidence qualitatively indicates a similar clinical response (relative PASI) to first and second line treatment, with chance of a good response up to fourth line treatment on a case‐by‐case basis. Meta‐analysis of PASI75/90/100 at 12–16 weeks found no diminishment of response from 1st to 4th line, but has to be interpreted with caution in view of the low data quality. Further prospective studies are required.

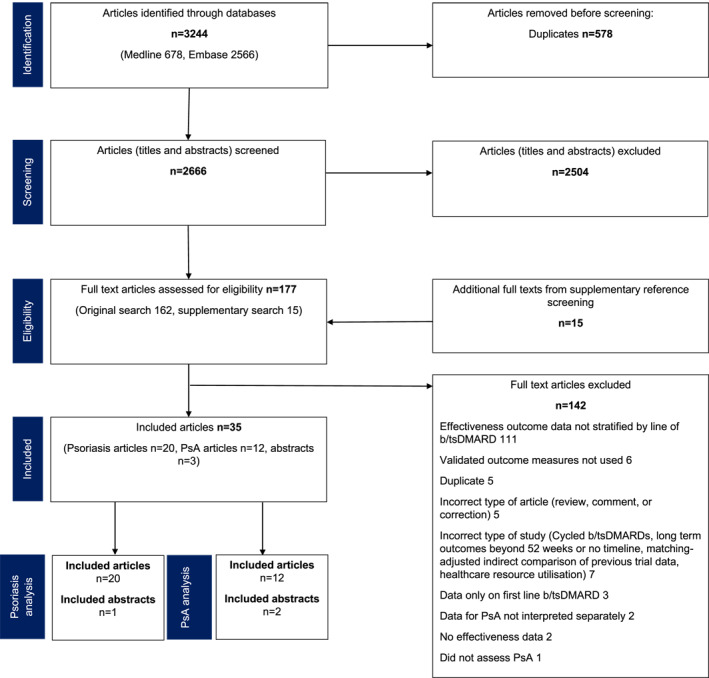

**What's already known about this topic?**

- Increasingly people with psoriasis are being treated with multiple sequential lines of biologic and targeted small molecule drugs.
- Drug survival has been shown to reduce with 2nd and beyond lines of biologic in psoriasis.
- First line biologic treatment in psoriasis has been shown to be efficacious in clinical trials, but clinical response to lines beyond first line is unclear.

**What does this study add?**

- The current available evidence shows that clinical response (as recorded by relative PASI) to first‐ and second‐line treatment is similar, and a good response can be achieved up to fourth‐line biologic treatment.
- There is very little data currently available on response to third‐ and fourth‐ine biologics, with no data for 5th+ line.
- A meta‐analysis of PASI75/90/100 at 12–16 weeks found a maintenance of response from 1st to 4th line biologic treatment.

## 1 INTRODUCTION

Psoriasis affects 60 million people worldwide,^1^ with prevalence varying according to country, from 0.14% in East Asia to 1.99% in Australasia.^2^ Up to 30% of those with skin psoriasis develop psoriatic arthritis (PsA), a heterogenous inflammatory musculoskeletal condition.^3^ Psoriatic disease leads to significant morbidity, a reduction in work productivity and an overall decline in quality of life.^4^, ^5^, ^6^

Over the past 2 decades, rapid advances have been made in the understanding and treatment of these conditions; multiple novel drugs have been approved, which have significantly advanced the treatment outcomes of people with psoriasis.^7^ In the United Kingdom, there are currently 13 biologic and targeted small molecule drugs approved for use in psoriasis, within the classes anti‐TNFα (TNFi), anti‐interleukin‐17 (IL‐17i), anti‐interleukin‐12/23 p40 subunit (IL‐12/23i), anti‐interleukin‐23 p19 subunit (IL‐23i) and PDE‐4 inhibitors (PDE4i).

Despite the efficacy of these biologics in clinical trials, with real‐world use a significant percentage of patients stop treatment, and switch to another biologic, due to factors including inefficacy and adverse effects.^8^ Due to the chronicity of psoriasis, increasingly patients have multiple lines of treatment over time. Registry data has shown that drug survival reduces with increasing lines of therapy.^9^ Whilst drug survival is an important measure of real‐world use, it does not directly correspond to efficacy and can be limited by availability of a medication, patient and physician behavioural factors and adverse effects.^10^

Randomised controlled trials, although best placed to determine efficacy of a novel biologic drug when used first line, often provide limited data on response to later lines of biologic treatment. Eligibility criteria in some cases limit the number and class of previous biologics, and the proportion of participants exposed to >1 prior biologic to avoid enriching the cohort with participants who may be difficult to treat.^11^ In those trials that include biologic‐experienced populations, sub‐analyses assessing response stratified by number of previous lines of biologic are limited.

There is some observational data indicating advanced lines of biologic, beyond first‐line, are effective, suggesting that benefit can be obtained by appropriate treatment switching. However, in both psoriasis and PsA, the clinical response may be lower in later lines than with first line treatment.^12^, ^13^ Understanding how patients respond to later lines of treatment improves treatment decisions for people with psoriatic disease. The development of evidence of effectiveness beyond third line would also help inform healthcare systems that currently ration the permitted number of lines of biologic per patient.

In this review we set out to appraise the current available evidence for effectiveness of biologics and targeted small molecules when used beyond first‐line in psoriasis.

## 2 METHODS

### 2.1 Protocol

A prospective protocol was registered with PROSPERO (CRD42022365298); this literature review is reported in line with PRISMA (Preferred Reporting Items for Systematic reviews and Meta‐Analyses) guidelines. The literature review was planned to assess response in all psoriatic disease, however for feasibility and comprehensibility, the analysis and reports have been separated into psoriasis and PsA (Gollins et al, under review), with this report focusing on studies assessing psoriasis only. Studies were included of adult patients (≥18 years) with psoriasis who had been treated with second and later line biologics and targeted small molecules, where the line of treatment is clearly established within the study and primary response was reported. We excluded studies of conventional systemic treatments.

### 2.2 Search strategy

The literature search was carried out on 10 October 2022 in Medline and 21 October 2022 in Embase. The search was repeated on 19 December 2022 prior to full analysis of included texts. Conference abstracts were included. The search included terms for psoriasis, PsA, biologic and targeted small molecule drugs, and lines of therapy (Appendix S1). There was no date limit. A supplementary manual search of bibliographies of cited articles was undertaken. Observational studies and randomised controlled trials were included if in the English language, or with an English language translation available. Papers reporting only drug persistence and not effectiveness for different lines of treatment were excluded. Randomised controlled trials and observational studies that assessed only first line biologics and targeted small molecules were excluded.

### 2.3 Selection of studies

All titles and abstracts from the searches were screened by two independent reviewers (CG, RV). Following this, full text review of selected papers was completed by one reader (CG). A further reader (RV) assessed all included full texts independently to confirm appropriateness. Any discrepancies, if not resolved, were resolved by a third senior reader (WT) at all levels of screening. CG completed data extraction, RV independently reviewed data extraction.

### 2.4 Data collection process and bias and quality assessment

Management of citations was undertaken with Refworks™ and Microsoft™ Excel software. A pre‐determined table was followed to extract data from included papers. Extracted data included country, year and type of study, average age and sex of study participants, sample size, intervention, class of intervention and prior biologics and targeted small molecules (before intervention drug). Time of follow up was limited to a maximum of 12 months, as the aim of the review was to assess primary or initial clinical response. Up to three time points per study were collected for feasibility, with the earliest being the closest to first follow up (typically 12–16 weeks) and the latest the closest time point to 12 months, if applicable. Rate of drop out was included where available. No assumptions were made regarding missing data.

The outcome assessed was Psoriasis Area and Severity Index (PASI), in particular 75%, 90% and 100% relative improvement outcomes (PASI75/90/100 respectively). Absolute PASI improvement was included where provided.

The methodological quality of the studies was assessed using the Cochrane Risk Of Bias In Non‐randomized Studies—of Interventions (ROBINS‐I) tool for all observational studies,^14^ and the Cochrane Risk of Bias tool 2 for all randomised controlled trials.^15^ Risk of bias assessments were not undertaken for abstracts due to lack of evidence available to complete a meaningful assessment. The quality of evidence was assessed by the Grading of Recommendations, Assessment, Development and Evaluation (GRADE) criteria for all studies included in the PASI75 meta‐analysis. There was a high level of crossover with studies included in the PASI90/100 meta‐analyses and so these studies were chosen to be representative of studies included in the statistical analyses.

### 2.5 Analysis

Studies were separated into manuscripts (Table S1) and abstracts (Table S2) for analysis. Manuscripts were narratively summarised, and statistically analysed as described. Abstracts were narratively summarised separately and were not included in the meta‐analysis, due to the limited available information prohibiting bias and quality assessment.

For manuscripts, a meta‐analysis was completed for the proportion of patients achieving PASI75, PASI90 and PASI100 at 12–16 weeks in each separate line of biologic treatment, for studies in which this was reported. These outcome measures were chosen as the most frequently reported within the included studies.

A proportional meta‐analysis using a random effects model was undertaken in MedCalc ™ for each group (1st, 2nd, 3rd/3rd + and 4th/4th + line biologic), with data included from all studies in which these outcomes were available. Abstracts were excluded from meta‐analysis. A random effects model was chosen due to the heterogeneity between studies, as indicated by the moderate to high I2 statistic found with each group of studies (Appendix S2).

A funnel plot was not undertaken to assess for reporting bias, as this test was primarily developed for comparative data, and is not recommended for use with proportional data.^16^

## 3 RESULTS

### 3.1 Study selection

**FIGURE 1:**
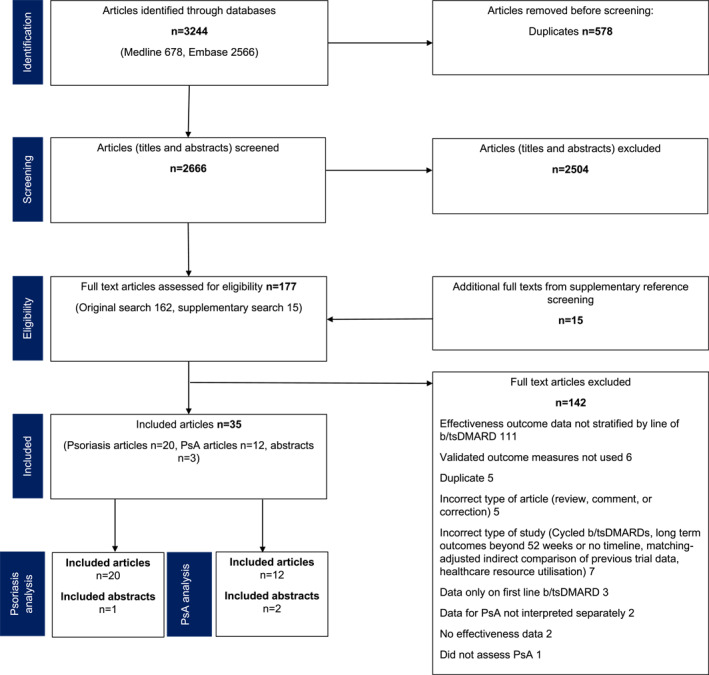
Study selection flow diagram for systematic review.

The search yielded 2666 unique papers and abstracts after duplicates had been removed (Figure 1) of which 162 were selected for full text screening. Fifteen additional papers were selected via bibliographic reference screening. Twenty manuscripts and one abstract assessing psoriasis were included.

Studies were excluded if lines of biologic were aggregated, for example, comparing ‘biologic‐naïve’ to ‘biologic experienced’ patients.^17^, ^18^ Several identified studies assessed response after an intra‐ or inter‐class switch of biologic, however were excluded from analysis if the prior number of lines of therapy were not clearly defined.^19^, ^20^ Both types of study described preclude analysis of effectiveness of specific lines of biologic and so were not appropriate for the current review. No contact with authors was required for clarification.

### 3.2 Study characteristics

Twenty studies meeting inclusion criteria were included (Table S1), published between 2007 and 2021, with data accrual from 2002 to 2019. Fifteen were based in Europe,^21^, ^22^, ^23^, ^24^, ^25^, ^26^, ^27^, ^28^, ^29^, ^30^, ^31^, ^32^, ^33^, ^34^, ^35^ two in Canada,^36^, ^37^ two in both Europe and North America^38^, ^39^ and one in Japan.^40^ As expected, the majority were observational studies (19/20) of which 11 were retrospective, and eight prospective. One sub‐analysis of a randomised controlled trial was included. There was a high rate of heterogeneity in study design and outcomes.

The percentage of patients achieving a given outcome for PASI75/90/100 and Physician Global Assessment are reported in Table S1. Mean PASI is displayed as an actual value, and mean PASI reduction as a percentage reduction from baseline.

### 3.3 Patient characteristics

**FIGURE 2:**
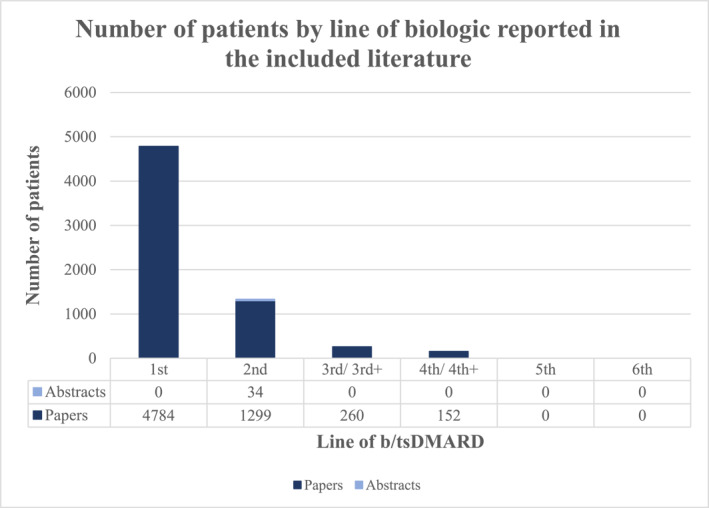
The number of patients reported in the included literature by line of biologic or targeted small molecule drug.

Included studies contained reported data from 6495 patients (Figure 2), with sample sizes varying considerably from 16 to 3038 patients' data per study. On average 35.1% were female (range 14%–54%) and mean age was 49.7 years (range 42–59).

### 3.4 Effectiveness of second‐line biologics in psoriasis

Eleven studies assessed the use of second‐line biologics and targeted small molecule drug, with or without a comparison to first‐line.^24^, ^25^, ^26^, ^27^, ^28^, ^29^, ^30^, ^31^, ^37^, ^38^, ^40^ Of the six studies that directly compared first‐to second‐line biologics or targeted small molecule drugs, four found comparable PASI outcomes in first‐ and second‐line.^26^, ^27^, ^31^, ^38^ The LIBERATE study^38^ was the only randomised controlled trial included, assessing apremilast versus placebo in biologic naïve patients, however also included an active comparator of etanercept. Patients within the active comparator line were switched to second‐line apremilast at 16 weeks, continuing to 52 weeks. PASI75 was similar in apremilast at week 52 in first‐line (53%) to week 36 in second‐line (57%), indicating similar PASI75 response for first‐ and second‐line biologic, however the study was not designed for first‐versus second‐line comparison.

In contrast, two studies found a higher PASI response in first‐line compared to second‐line,^30^, ^40^ with one study^40^ reaching significance (*p* < 0.05). This study compared first‐ and second‐line biologics in a single centre group of patients (*n* = 326). A higher rate of PASI75 was found in first‐than second‐line at 14–16 weeks (59.1% vs. 43.7%), but mean PASI was significantly lower in second‐line (2.9) than first‐line (4.3). This discrepancy, with a greater PASI75 response in first‐line, but a higher mean PASI at 14–16 weeks, was due to the lower baseline PASI at the start of second‐line treatment.

The further five studies assessed second‐line treatment only, with no lines of comparison^24^, ^25^, ^28^, ^29^, ^37^: results shown in Table S1.

### 3.5 Effectiveness of third‐line + biologics in psoriasis

Nine studies assessed third‐ and later line biologic or targeted small molecules.^21^, ^22^, ^23^, ^32^, ^33^, ^34^, ^35^, ^36^, ^39^ Four of the studies found a reduction in effectiveness in third‐ or fourth‐line, compared to first‐ or second‐line.^23^, ^33^, ^36^, ^39^ This included the largest study in this group,^39^ PSO‐BIO‐REAL (*n* = 846), which was a multi‐national prospective study of biologic‐naive and ‐experienced patients over 12 months, to assess for complete skin clearance. Generally, the rate of PASI100 decreased with increased numbers of prior biologics: 25% at week 26 for first‐line compared to 14% for fourth‐line.

A diminishing response with successive lines of biologic was not however found in all included studies, with some finding varying differences in outcomes at different time points. A multicentre study^21^ retrospectively assessed the use of risankizumab in 77 patients, finding the odds of achieving PASI75 were significantly lower in patients on 3rd+ line of biologic compared to first‐ and second‐line, at week 16 (*p* 0.018) however there was no difference between lines in achieving PASI75 at week 40. The real‐world use of secukinumab in two centres was retrospectively assessed,^22^ with mean PASI reported as lower at week 12 with third‐line treatment (3.8) than first‐(5.3) and second‐line (4.6), but higher by week 40 (3.7 vs. 1.3 and 2.4).

Furthermore, two studies found a reduction in effectiveness (PASI75/90/100) beyond first‐line, but no difference between subsequent lines^32^, ^35^ and Papoutsaki et al^34^ found a higher PASI90 at 24 weeks in fourth‐than third‐line adalimumab (100% vs. 63%) in a small open label single‐centre study. Notably, the frequency of adalimumab used was higher than currently licenced for psoriasis (40 mg weekly) which may explain the high effectiveness compared to other studies.

### 3.6 Comparison of studies

**FIGURE 4:**
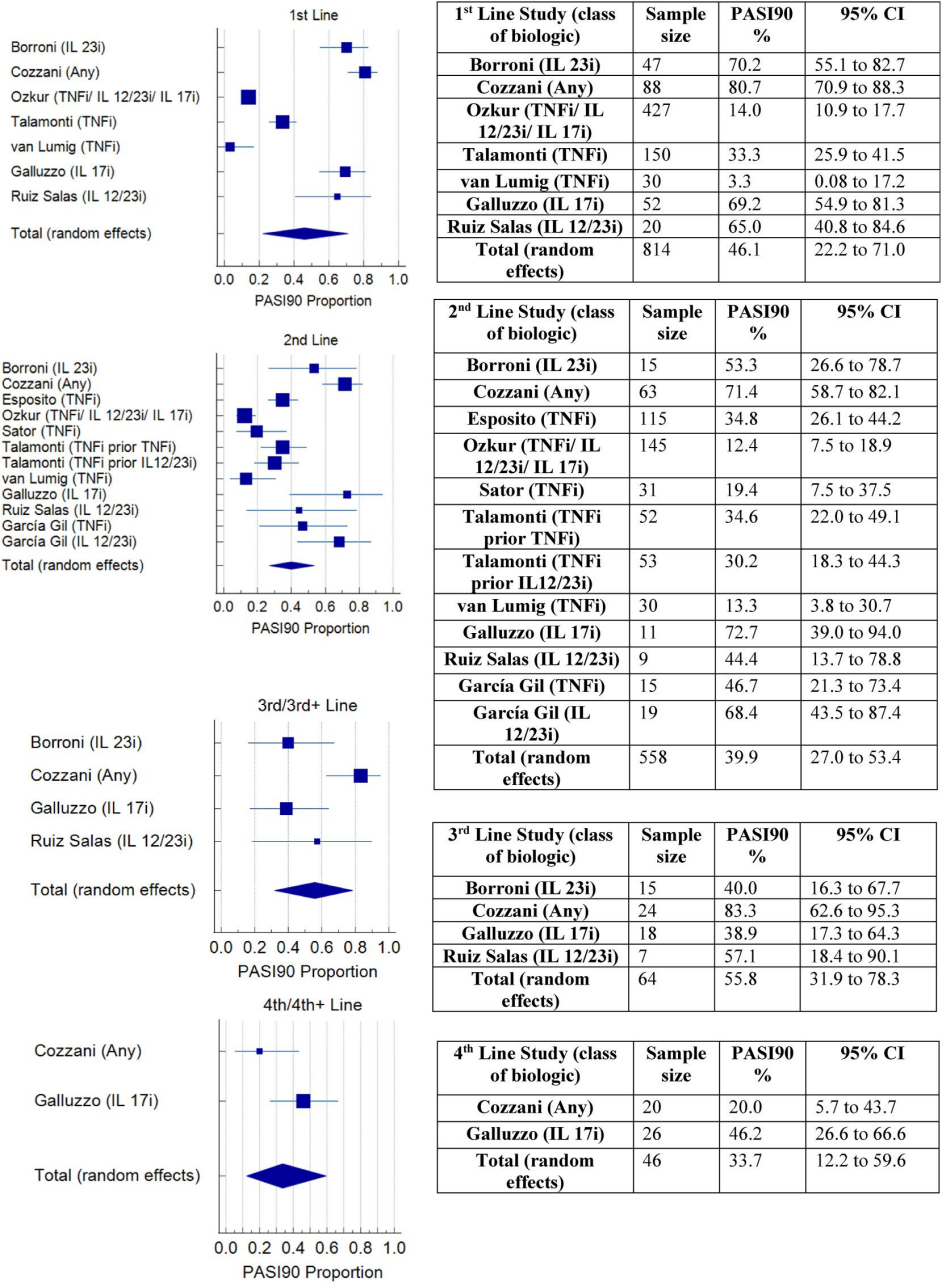
Meta‐analysis of PASI90 at 12–16 weeks for 1st to 4th line.

**FIGURE 5:**
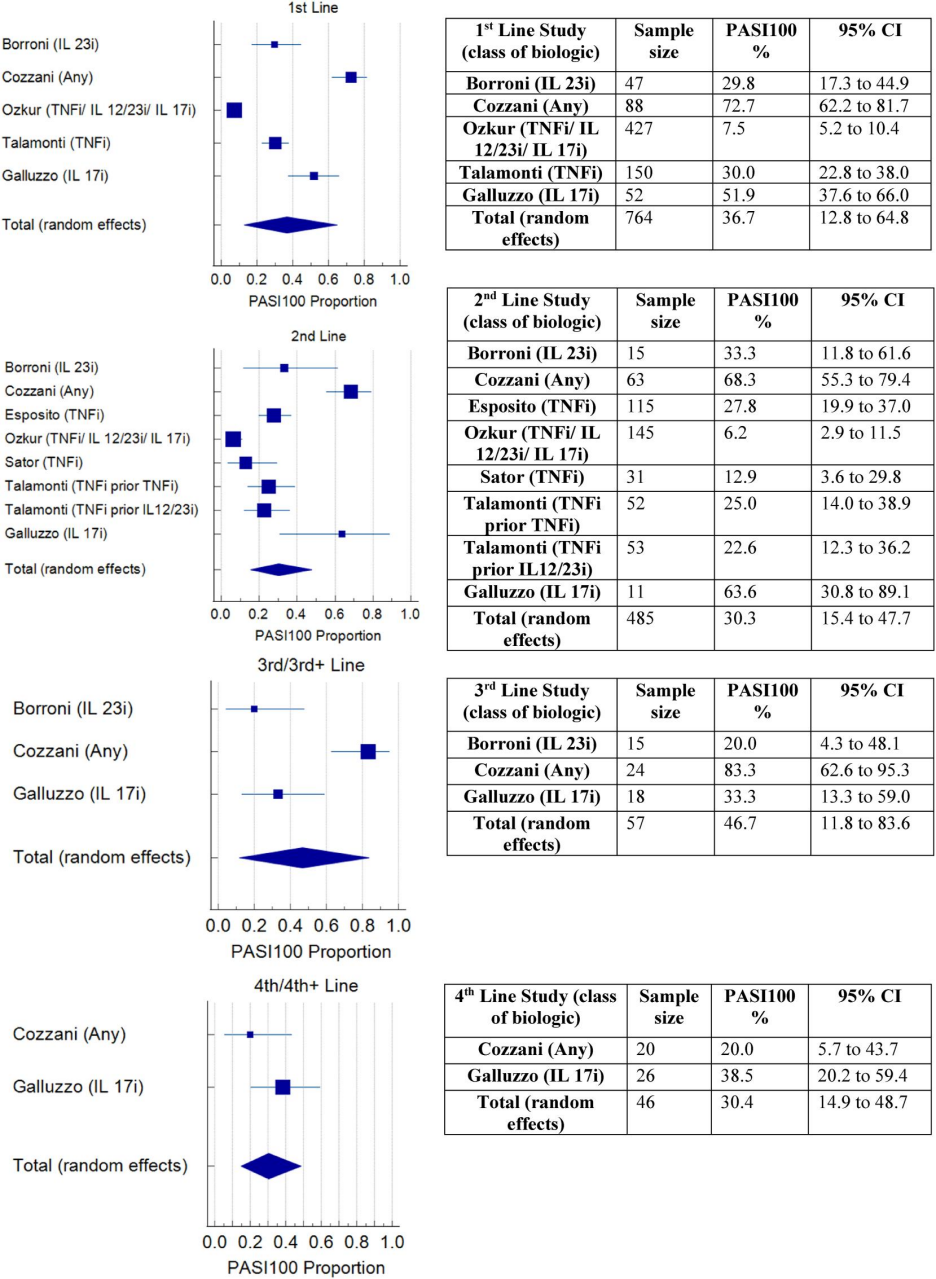
Meta‐analysis of PASI100 at 12–16 weeks for 1st–4th line.

A meta‐analysis of the proportion of participants achieving PASI75 at 12–16 weeks, in the studies that recorded this outcome (16/20), found that effectiveness did not appear to reduce with successive lines of biologic (Figure 3). The pooled effect percentage of patients achieving PASI75 with 1st, 2nd, 3rd and 4th line biologics was 61%, 56%, 79% and 61% respectively. A meta‐analysis of PASI90 at 12–16 weeks in studies that included this outcome (10/20) found a pooled effect percentage achieving PASI90 of 46.1%, 39.9%, 55.8% and 33.7% in 1st to 4th line respectively (Figure 4). A meta‐analysis of PASI100 at 12–16 weeks (7 studies) found a pooled effect percentage achieving PASI100 of 36.7%, 30.3%, 46.7% and 30.4% in 1st to 4th line biologics respectively (Figure 5).

FIGURE 3(a) Meta‐analysis of PASI75 at 12–16 weeks for 1st and 2nd line. (b) Meta‐analysis of PASI75 at 12–16 weeks for 3rd and 4th line.

**Figure:**
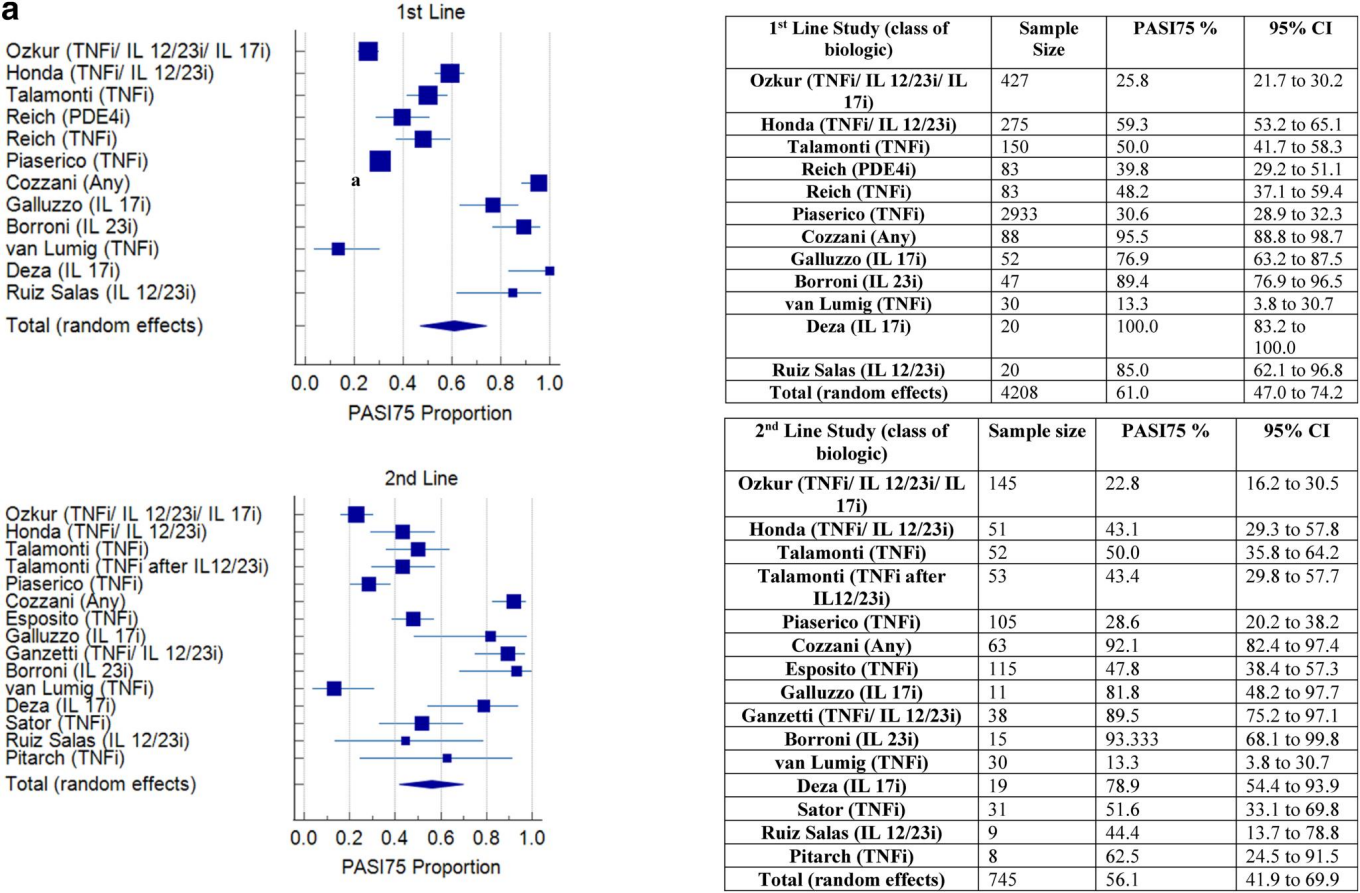

**Figure:**
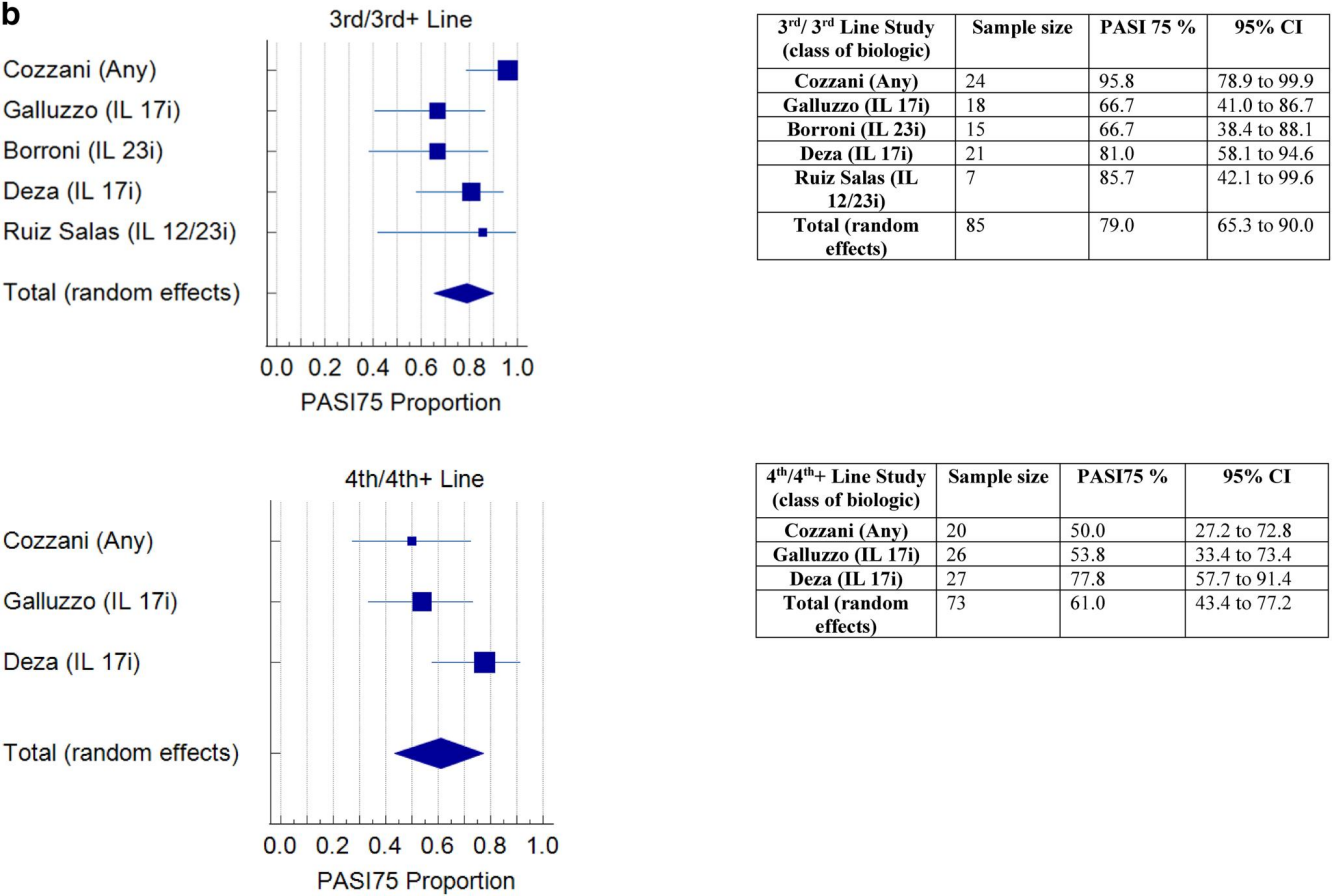

### 3.7 Abstracts

Only one abstract of a small retrospective European study of 34 patients met inclusion criteria^41^; results displayed in Table S2. This study assessed PASI90 response to second‐line adalimumab or ustekinumab after etanercept. A numerically but not statistically higher response was found in second line ustekinumab (PASI90 68.4%) compared to adalimumab (PASI90 46.6%) at 12 weeks.

### 3.8 Bias and quality assessment

Risk of bias was assessed for all included studies (Tables 1 and 2), with the overall risk of bias considered ‘serious’ or ‘of some concern’ for all studies. ROBINS‐I assesses bias by comparing an observational study to a ‘target randomised controlled trial’, or the ideal RCT that could answer the research question, whether or not this would be feasible in the real world. It is therefore inevitable that most observational studies will score seriously for bias, due to inherent limitations in study design when compared to an RCT, for example, selection bias. The GRADE criteria confirmed that the available evidence is of very low quality (Table 3). It is therefore important to interpret the meta‐analysis results with caution. However, these studies currently provide the best evidence available for understanding effectiveness of advanced lines of treatment in psoriasis.

**TABLE 1:** Assessment of bias for included observational studies utilising the ROBINS‐I assessment tool.

Study	Bias due to confounding	Bias in selection of participants	Bias in classification of interventions	Bias due to deviations from intended intervention	Bias due to missing data	Bias in measurement of outcomes	Bias in selection of reported result	Overall risk of bias
Borroni 2021	Serious	Serious	Low	No information	Moderate	Serious	Serious	Serious
Carpentieri 2020	Serious	Serious	Low	No information	No information	Serious	Serious	Serious
Cozzani 2020	Serious	Serious	Serious	No information	No information	Serious	Serious	Serious
Deza 2018	Serious	Serious	Moderate	No information	Serious	Serious	Serious	Serious
Esposito 2019	Serious	Serious	Moderate	Serious	No information	Serious	Serious	Serious
Galluzzo 2018	Serious	Serious	Low	No information	Serious	Serious	Serious	Serious
Ganzetti 2018	Serious	Serious	Moderate	No information	No information	Serious	Serious	Serious
Honda 2017	Serious	Serious	Serious	No information	No information	Serious	Serious	Serious
Ozkur 2021	Serious	Serious	No information	Serious	No information	Serious	Serious	Serious
Papoutsaki 2007	Serious	Serious	Low	No information	No information	Serious	Serious	Serious
Piaserico 2014	Serious	Serious	Moderate	No information	Low	Serious	Serious	Serious
Pitarch 2008	Serious	Serious	Moderate	Serious	Moderate	Serious	Serious	Serious
Qiang 2016	Serious	Serious	Serious	No information	No information	Serious	Serious	Serious
Ruiz Salas 2012	Serious	Serious	Low	Moderate	Low	Serious	Serious	Serious
Sator 2015	Serious	Serious	Moderate	Serious	Serious	Serious	Low	Serious
Seneschal 2020	Serious	Serious	Serious	No information	No information	Serious	Serious	Serious
Talamonti 2018	Moderate	Serious	Moderate	No information	Moderate	Serious	Serious	Serious
van Lumig 2010	Serious	Serious	Serious	Moderate	Serious	Serious	Serious	Serious
Vender 2011	Serious	Serious	Low	No information	No information	Serious	Low	Serious

**TABLE 2:** Assessment of bias for included sub‐analyses of randomised controlled trials utilising the Cochrane Risk of Bias 2 tool.

Study	Bias arising from randomization process	Bias due to deviations from intended interventions	Bias due to missing outcome data	Bias in measurement of outcome	Bias in selection of reported result	Overall risk of bias
Reich 2017	Low	Low	Some concerns	Low	Some concerns	Some concerns

**TABLE 3:** Assessment of quality of eligible studies in PASI75 meta‐analysis, separated by line of biologic or targeted small molecule using GRADE criteria.

Quality assessment of included studies in meta‐analysis of PASI75								Effect		Quality
Number of studies	Design of studies	Risk of bias	Inconsistency	Indirectness	Imprecision	Other considerations	Number of patients included	Absolute (PASI75%)	Relative (95% CI)	
First line										
11	11 NRSI	Serious^a^	Some inconsistency^b^	Direct	No serious imprecision	None	4208	61.0	47.0–74.2	Very low
	1 RCT sub‐analysis									⊕ΟΟΟ
Second line										
14	14 NRSI	Serious^a^	Some inconsistency^b^	Direct	No serious imprecision	None	745	56.1	41.9–69.9	Very low
										⊕ΟΟΟ
Third/third + line										
5	5 NRSI	Serious^a^	Some inconsistency^c^	Direct	Some imprecision^d^	None	85	79.0	65.3–90.0	Very low
										⊕ΟΟΟ
Fourth/fourth + line										
3	3 NRSI	Serious^a^	Some inconsistency^c^	Direct	Some imprecision^d^	None	73	61.0	43.4–77.3	Very low
										⊕ΟΟΟ

Abbreviations: CI, confidence intervals; NRSI, non‐randomised studies on interventions; PASI, psoriasis area severity index; RCT, randomised controlled trials.

^a^ Details of risk of bias of all studies present in Table 3.

^b^ Significant statistical heterogeneity and wide variation in effect estimates across studies.

^c^ Moderate statistical heterogeneity.

^d^ small sample size.

## 4 DISCUSSION

The overall aim of this systematic review was to evaluate the evidence of primary response of lines of biologics and targeted small molecules in psoriasis, beyond first line.

In qualitative assessment of eligible studies, PASI response to first‐ and second‐line biologics is often similar, but there is a variation in response to third‐line and beyond (Appendix S3). A meta‐analysis of PASI75/90/100 did not find a reduction in response to sequential lines of biologic/targeted small molecule beyond first line for all three outcome measures. In fact, pooled response to third line exceeded response to second line in all assessed outcome measures. However there was significant heterogeneity found within the studies, as well as very low numbers of included cases in the analysis of 3rd and 4th lines. It is possible that there was publication bias in the studies reporting later lines of therapy, leading to higher than expected responses to 3rd and 4th lines. The low sample sizes, heterogeneity and low quality of the studies limit the applicability of the meta‐analysis results.

The review only included studies assessing second and later lines of treatment, and therefore the assessment of response to first line is taken entirely from eligible studies that had a first line comparator. It is likely that if studies assessing first line alone had been included, within this line, bias would be lower and quality higher as a first line biologics would have been assessed in the context of randomised controlled trials.

There is overall very little data available at third‐line and beyond, with only 412 patients contributing to results at third‐ and fourth‐line, and no current evidence in the literature for response to biologics or targeted small molecules at 5th+ lines. Lower numbers of patients assessed in later lines reduced the power of eligible observational studies, often precluding statistical testing to assess for differences in outcome compared to first‐ and second‐line.

Relative PASI (PASI75/90/100) is conventionally accepted as the primary outcome measure for efficacy of psoriasis drugs. However it has been suggested that absolute PASI, for example, PASI < 3 or ≤ 2, is an additional relevant disease end point.^40^, ^42^, ^43^ Relative PASI may be less appropriate to assess later lines of treatment, as baseline PASI at the start of successive lines of biologic tends to decrease, potentially leading to more difficulty in achieving relative PASI scores.^40^, ^43^ This could have played a role in the included studies due to the lack of wash out periods in most. This would lead to disease activity improvement from one line of treatment being carried over to the baseline of the next drug, particularly in cases where the drug was stopped due to factors other than efficacy, for example, adverse effects.

The review is limited to 12 months follow up, to focus on primary response, hence long‐term effectiveness is not assessed.

## 5 CONCLUSION

The current evidence available for effectiveness of advanced lines of biologic and targeted small molecule therapy for the treatment of psoriasis is primarily observational, at high risk of bias and of very low quality. Qualitatively, response in psoriasis to first‐ and second‐line biologics appears similar, with a reduction in response from third‐line suggested by individual studies but not confirmed by meta‐analysis. Meta‐analysis of PASI75/90/100 did not find a diminishment in effectiveness with sequential lines of treatment, but needs to be interpreted with caution. There is good evidence of a potential benefit to patients in later lines of treatment on a case‐by‐case basis. Further prospective studies are required to build an understanding of response to third‐line and beyond biologics in psoriasis.

## AUTHOR CONTRIBUTIONS

**Charlotte E. Gollins**: Conceptualization (equal); data curation (lead); formal analysis (lead); methodology (equal); project administration (lead); writing – original draft (lead); writing – review & editing (lead). **Rosie Vincent**: Data curation (equal); formal analysis (equal); methodology (equal); writing – review & editing (equal). **Caoimhe Fahy**: Conceptualization (equal); methodology (equal); supervision (equal); writing – review & editing (equal). **Neil McHugh**: Conceptualization (equal); formal analysis (equal); methodology (equal); supervision (equal); visualization (equal); writing – review & editing (equal). **William Tillett**: Conceptualization (equal); methodology (equal); supervision (lead); visualization (equal); writing – review & editing (equal).

## CONFLICT OF INTEREST STATEMENT

WRT has received research funding, speaker fees or honoraria from Abbvie, Amgen, Eli‐Lilly, GSK, Janssen, MSD, Novartis, Ono‐Pharma, Pfizer and UCB. CF has received honararia (speaker fees) from Pfizer and Eli Lilly. NJM has received a grant for unrelated work from UCB, honoraria (speaker fee) from Janssen and participated in data monitoring and safety in the NIHR HTA Astute trial. RV has received payment to her institution (speaker fees) from Leo Pharma and funding from Dermal to attend an educational event. CG has no conflicts of interest to declare.

## ETHICS STATEMENT

Not applicable.

## Supporting information

Supporting Information S1

Supporting Information S2

Supporting Information S3

Table S1

Table S2

## Data Availability

No new data were generated or analysed in support of this research.
