# Supplementary material for: Effectiveness of sequential lines of biologic and targeted small molecule drugs in psoriasis: A systematic review and meta‐analysis
Source: Skin Health Dis. 2024 Feb 29;4(2):e350. doi: 10.1002/ski2.350 (PMC10988728; doi:10.1002/ski2.350)
Supplement: Supplementary file 1 — Supporting Information S1 [file SKI2-4-e350-s002.docx]

**Supplementary Material 1: Medline and Embase Search Strategies**

**Ovid MEDLINE(R) <1946 to December Week 2 2022>**

1 exp Psoriasis/ 46614

2 exp Psoriasis/dt [Drug Therapy] 19156

3 exp Arthritis, Psoriatic/ 7745

4 psoriasis.ab,ti. 41636

5 1 or 4 54270

6 2 or 4 46422

7 exp Biological Products/ 660225

8 exp Biological Factors/ 3855404

9 "biologic*".ab,ti. 884525

10 "tnf inhibitor* ".ab,ti. 1893

11 interleukin-17 inhibitor.ab,ti. 19

12 interleukin-23 inhibitor.ab,ti. 11

13 "interleukin 12/23 inhibitor".ab,ti. 17

14 Il-17.ab,ti. 18012

15 Il-23.ab,ti. 5964

16 "il-12/ 23".ab,ti. 408

17 pde4.ab,ti. 2260

18 jak inhibitor.ab,ti. 1080

19 exp Tumor Necrosis Factor Inhibitors/ 17599

20 exp Interleukin-17/ 14037

21 exp Interleukin-23/ 4635

22 exp Interleukin-12/ 15122

23 exp Phosphodiesterase 4 Inhibitors/ 2585

24 exp Janus Kinase Inhibitors/ 1276

25 7 or 8 or 9 or 10 or 11 or 12 or 13 or 14 or 15 or 16 or 17 or 18 or 19 or 20 or 21 or 22 or 23 or 24 4995108

26 5 and 25 20767

27 6 and 25 18873

28 7 or 9 or 10 or 11 or 12 or 13 or 14 or 15 or 16 or 17 or 18 or 19 or 20 or 21 or 22 or 23 or 24 1558998

29 5 and 28 10876

30 29 10876

31 limit 30 to english language 10291

32 2nd line.ab,ti. 359

33 second-line.ab,ti. 23389

34 3rd line.ab,ti. 180

35 third-line.ab,ti. 4166

36 4th line.ab,ti. 57

37 fourth-line.ab,ti. 610

38 5th line.ab,ti. 19

39 fifth-line.ab,ti. 141

40 6th line.ab,ti. 12

41 sixth-line.ab,ti. 49

42 sequential*.ab,ti. 163605

43 first-line.ab,ti. 85823

44 1st line.ab,ti. 453

45 32 or 33 or 34 or 35 or 36 or 37 or 38 or 39 or 40 or 41 or 42 or 43 or 44 264401

46 31 and 45 292

47 psoriatic arthritis.ab,ti. 9383

48 3 or 47 10798

49 25 and 48 4918

50 45 and 49 144

51 50 144

52 limit 51 to english language 139

53 46 or 52 328

54 switch.ab,ti. 72554

55 switching.ab,ti. 51307

56 45 or 54 or 55 375089

57 Adalimumab.ab,ti. 7019

58 Apremilast.ab,ti. 707

59 Bimekizumab.ab,ti. 54

60 Brodalumab.ab,ti. 330

61 Certolizumab.ab,ti. 1020

62 Deucravacitinib.ab,ti. 13

63 Etanercept.ab,ti. 6767

64 Guselkumab.ab,ti. 343

65 Infliximab.ab,ti. 12030

66 Ixekizumab.ab,ti. 608

67 Netakimab.ab,ti. 8

68 Risankizumab.ab,ti. 218

69 Secukinumab.ab,ti. 1229

70 Sonelokimab.ab,ti. 2

71 Tildrakizumab.ab,ti. 160

72 Ustekinumab.ab,ti. 2047

73 Upadacitinib.ab,ti. 259

74 Tofacitinib.ab,ti. 1730

75 Golimumab.ab,ti. 1086

76 exp Infliximab/ 11760

77 exp Etanercept/ 6418

78 exp Certolizumab Pegol/ 721

79 exp Adalimumab/ 6683

80 "biologic experienced".ab,ti. 60

81 "bio-experienced".ab,ti. 15

82 "anti-TNF experienced".ab,ti. 24

83 "TNF experienced".ab,ti. 28

84 "anti-TNF-IR".ab,ti. 9

85 28 or 57 or 58 or 59 or 60 or 61 or 62 or 63 or 64 or 65 or 66 or 67 or 68 or 69 or 70 or 71 or 72 or 73 or 74 or 75 or 76 or 77 or 78 or 79 or 80 or 81 or 82 or 83 or 84 1566871

86 5 and 85 12830

87 56 and 86 694

88 87 694

89 limit 88 to english language 678

**Embase <1974 to 2022 December 19>**

1 exp Psoriasis/ 105135

2 exp Psoriasis/dt [Drug Therapy] 31084

3 exp Arthritis, Psoriatic/ 29011

4 psoriasis.ab,ti. 71124

5 1 or 4 111308

6 2 or 4 82728

7 exp Biological Products/ 827024

8 exp Biological Factors/ 3278556

9 "biologic*".ab,ti. 1267857

10 "tnf inhibitor* ".ab,ti. 5748

11 interleukin-17 inhibitor.ab,ti. 54

12 interleukin-23 inhibitor.ab,ti. 43

13 "interleukin 12/23 inhibitor".ab,ti. 37

14 Il-17.ab,ti. 34523

15 Il-23.ab,ti. 11326

16 "il-12/ 23".ab,ti. 944

17 pde4.ab,ti. 3541

18 jak inhibitor.ab,ti. 2963

19 exp Tumor Necrosis Factor Inhibitors/ 114540

20 exp Interleukin-17/ 61947

21 exp Interleukin-23/ 18086

22 exp Interleukin-12/ 54088

23 exp Phosphodiesterase 4 Inhibitors/ 13338

24 exp Janus Kinase Inhibitors/ 26328

25 7 or 8 or 9 or 10 or 11 or 12 or 13 or 14 or 15 or 16 or 17 or 18 or 19 or 20 or 21 or 22 or 23 or 24 5020464

26 5 and 25 49389

27 6 and 25 38654

28 7 or 9 or 10 or 11 or 12 or 13 or 14 or 15 or 16 or 17 or 18 or 19 or 20 or 21 or 22 or 23 or 24 2221173

29 5 and 28 37775

30 29 37775

31 limit 30 to english language 36245

32 2nd line.ab,ti. 4148

33 second-line.ab,ti. 50978

34 3rd line.ab,ti. 1883

35 third-line.ab,ti. 10441

36 4th line.ab,ti. 575

37 fourth-line.ab,ti. 1539

38 5th line.ab,ti. 176

39 fifth-line.ab,ti. 367

40 6th line.ab,ti. 95

41 sixth-line.ab,ti. 129

42 sequential*.ab,ti. 241288

43 first-line.ab,ti. 178986

44 1st line.ab,ti. 5326

45 32 or 33 or 34 or 35 or 36 or 37 or 38 or 39 or 40 or 41 or 42 or 43 or 44 455899

46 31 and 45 1194

47 psoriatic arthritis.ab,ti. 22701

48 3 or 47 31081

49 25 and 48 17297

50 45 and 49 654

51 50 654

52 limit 51 to english language 640

53 46 or 52 1230

54 switch.ab,ti. 116289

55 switching.ab,ti. 90691

56 45 or 54 or 55 637218

57 Adalimumab.ab,ti. 22328

58 Apremilast.ab,ti. 1915

59 Bimekizumab.ab,ti. 184

60 Brodalumab.ab,ti. 675

61 Certolizumab.ab,ti. 4011

62 Deucravacitinib.ab,ti. 70

63 Etanercept.ab,ti. 16522

64 Guselkumab.ab,ti. 965

65 Infliximab.ab,ti. 30281

66 Ixekizumab.ab,ti. 1648

67 Netakimab.ab,ti. 49

68 Risankizumab.ab,ti. 521

69 Secukinumab.ab,ti. 3634

70 Sonelokimab.ab,ti. 6

71 Tildrakizumab.ab,ti. 445

72 Ustekinumab.ab,ti. 6123

73 Upadacitinib.ab,ti. 993

74 Tofacitinib.ab,ti. 4903

75 Golimumab.ab,ti. 4535

76 exp Infliximab/ 59896

77 exp Etanercept/ 36333

78 exp Certolizumab Pegol/ 8689

79 exp Adalimumab/ 42594

80 "biologic experienced".ab,ti. 367

81 "bio-experienced".ab,ti. 94

82 "anti-TNF experienced".ab,ti. 138

83 "TNF experienced".ab,ti. 156

84 "anti-TNF-IR".ab,ti. 62

85 28 or 57 or 58 or 59 or 60 or 61 or 62 or 63 or 64 or 65 or 66 or 67 or 68 or 69 or 70 or 71 or 72 or 73 or 74 or 75 or 76 or 77 or 78 or 79 or 80 or 81 or 82 or 83 or 84 2224753

86 5 and 85 39466

87 56 and 86 2621

88 87 2621

89 limit 88 to english language 2566
