# Supplementary material for: Effectiveness of sequential lines of biologic and targeted small molecule drugs in psoriasis: A systematic review and meta‐analysis
Source: Skin Health Dis. 2024 Feb 29;4(2):e350. doi: 10.1002/ski2.350 (PMC10988728; doi:10.1002/ski2.350)
Supplement: Supplementary file 2 — Supporting Information S2 [file SKI2-4-e350-s004.docx]

**Appendix S2: Heterogeneity assessments for all meta-analyses**

**PASI75 Meta-analysis**

*1^st^ Line*

| **Q** | 501.2261 |
| --- | --- |
| **DF** | 11 |
| **Significance level** | P < 0.0001 |
| **I^2^ (inconsistency)** | 97.81% |
| **95% CI for I^2^** | 97.11 to 98.34 |

*2^nd^ Line*

| **Q** | 205.2803 |
| --- | --- |
| **DF** | 14 |
| **Significance level** | P < 0.0001 |
| **I^2^ (inconsistency)** | 93.18% |
| **95% CI for I^2^** | 90.33 to 95.19 |

*3^rd^ Line*

| **Q** | 8.4940 |
| --- | --- |
| **DF** | 4 |
| **Significance level** | P = 0.0751 |
| **I^2^ (inconsistency)** | 52.91% |
| **95% CI for I^2^** | 0.00 to 82.68 |

*4^th^ Line*

| **Q** | 4.9242 |
| --- | --- |
| **DF** | 2 |
| **Significance level** | P = 0.0853 |
| **I^2^ (inconsistency)** | 59.38% |
| **95% CI for I^2^** | 0.00 to 88.43 |

**PASI90 Meta-analysis**

*1^st^ Line*

| **Q** | 258.2920 |
| --- | --- |
| **DF** | 6 |
| **Significance level** | P < 0.0001 |
| **I^2^ (inconsistency)** | 97.68% |
| **95% CI for I^2^** | 96.60 to 98.41 |

*2^nd^ Line*

| **Q** | 106.0705 |
| --- | --- |
| **DF** | 11 |
| **Significance level** | P < 0.0001 |
| **I^2^ (inconsistency)** | 89.63% |
| **95% CI for I^2^** | 83.82 to 93.35 |

*3^rd^ Line*

| **Q** | 11.7627 |
| --- | --- |
| **DF** | 3 |
| **Significance level** | P = 0.0082 |
| **I^2^ (inconsistency)** | 74.50% |
| **95% CI for I^2^** | 28.96 to 90.84 |

*4^th^ Line*

| **Q** | 3.3839 |
| --- | --- |
| **DF** | 1 |
| **Significance level** | P = 0.0658 |
| **I^2^ (inconsistency)** | 70.45% |
| **95% CI for I^2^** | 0.00 to 93.35 |

**PASI100 Meta-analysis**

*1^st^ Line*

| **Q** | 202.7331 |
| --- | --- |
| **DF** | 4 |
| **Significance level** | P < 0.0001 |
| **I^2^ (inconsistency)** | 98.03% |
| **95% CI for I^2^** | 96.94 to 98.73 |

*2^nd^ Line*

| **Q** | 102.9175 |
| --- | --- |
| **DF** | 7 |
| **Significance level** | P < 0.0001 |
| **I^2^ (inconsistency)** | 93.20% |
| **95% CI for I^2^** | 88.89 to 95.84 |

*3^rd^ Line*

| **Q** | 19.7656 |
| --- | --- |
| **DF** | 2 |
| **Significance level** | P = 0.0001 |
| **I^2^ (inconsistency)** | 89.88% |
| **95% CI for I^2^** | 72.89 to 96.22 |

*4^th^ Line*

| **Q** | 1.7524 |
| --- | --- |
| **DF** | 1 |
| **Significance level** | P = 0.1856 |
| **I^2^ (inconsistency)** | 42.94% |
| **95% CI for I^2^** | 0.00 to 0.00 |
