# Supplementary material for: Effectiveness of sequential lines of biologic and targeted small molecule drugs in psoriasis: A systematic review and meta‐analysis
Source: Skin Health Dis. 2024 Feb 29;4(2):e350. doi: 10.1002/ski2.350 (PMC10988728; doi:10.1002/ski2.350)
Supplement: Supplementary file 3 — Supporting Information S3 [file SKI2-4-e350-s001.pdf]

Appendix S3: Graphs to show intra- and inter-study comparative PASI75/90/100 at 12-16 weeks in 1st to 4th line biologic and targeted small molecule treatment in included studies that recorded these outcome measures.

PASI75 at 12-16 weeks

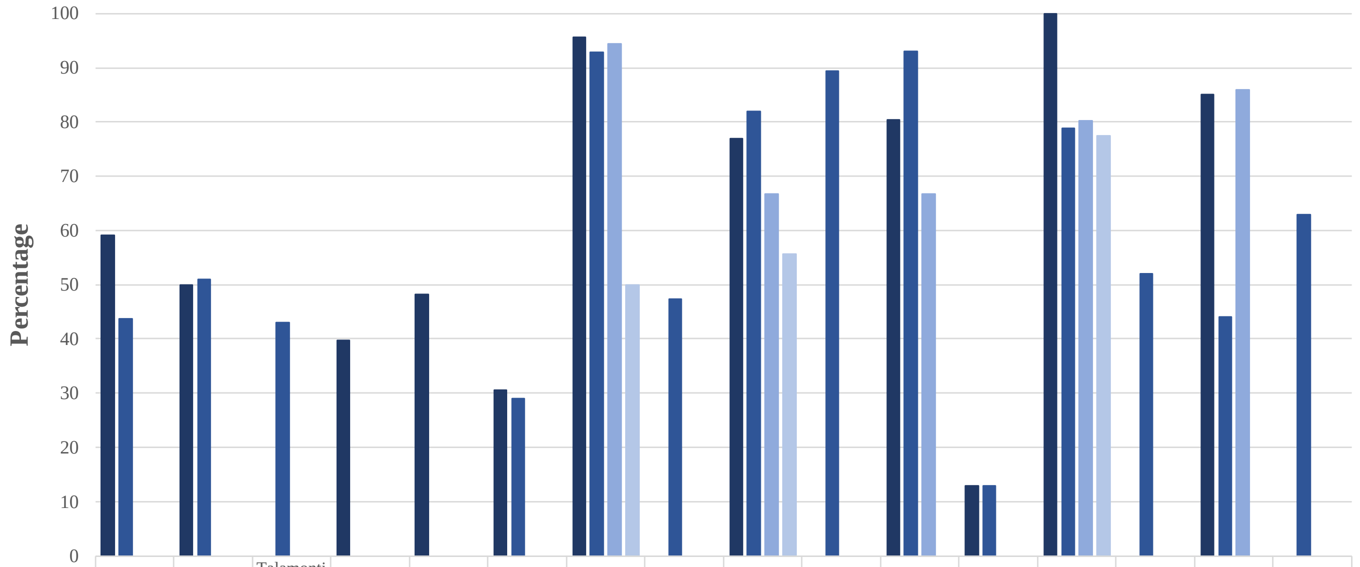

	Honda (TNF/ IL 12/23)	Talamonti (TNF only)	Talamonti (TNF after IL12/23)	Reich (PDE4)	Reich (TNF)	Piaserico (TNF)	Cozzani (Any)	Esposito (TNF)	Galluzzo (IL 17)	Ganzetti (TNF/ IL 12/23	Borroni (IL 23)	van Lumig (TNF)	Deza (IL 17)	Sator (TNF)	Ruiz Salas (IL 12/23)	Pitarch (TNF)
1st Line	59.1	50.0		39.8	48.2	30.6	95.7		76.9		80.4	13.0	100.0		85.0	
2nd Line	43.7	51	43			29	92.9	47.4	81.9	89.4	93	13	78.9	52	44	63
3rd Line							94.4		66.7		66.7		80.3		86	
4th Line							50		55.6				77.5			

Study (Class of b/tsDMARD)

1st Line 2nd Line 3rd Line 4th Line

PASI90 at 12-16 weeks

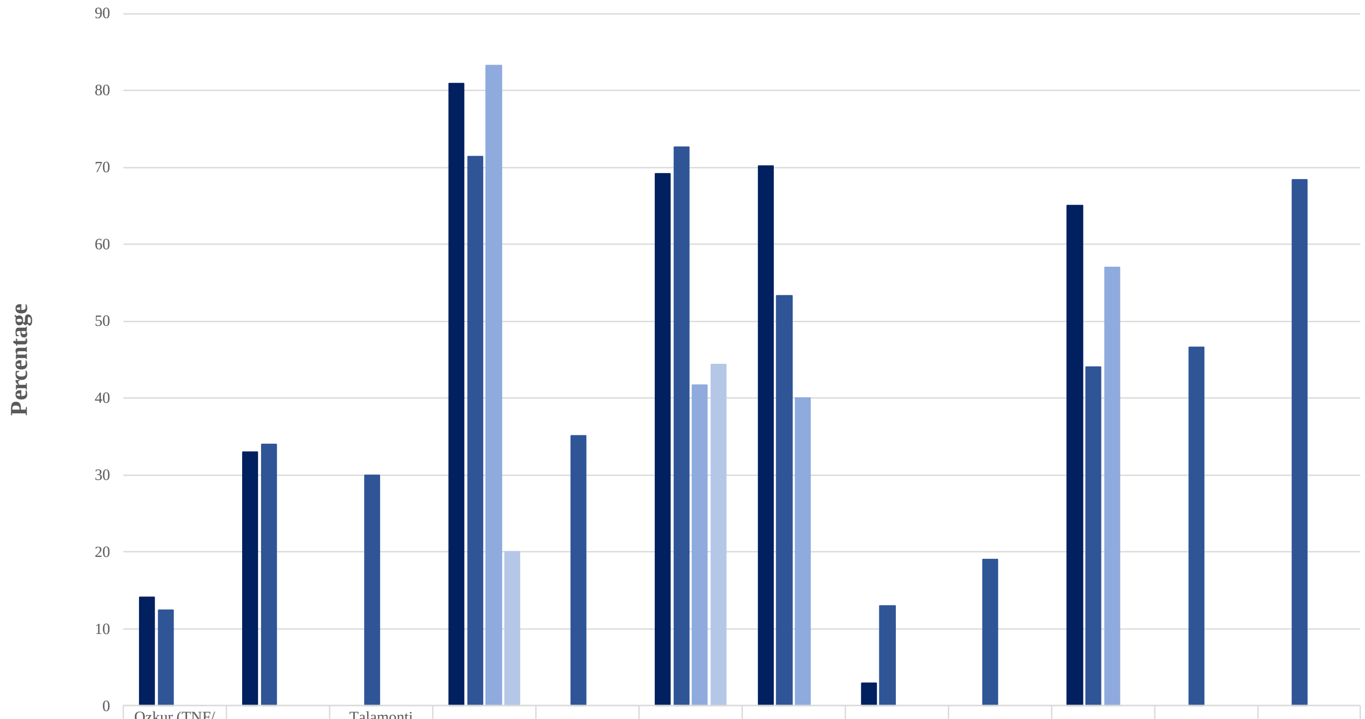

	Ozkur (TNF/ IL 12/23/ IL 17)	Talamonti (TNF only)	Talamonti (TNF after IL 12/23)	Cozzani (Any)	Esposito (TNF)	Galluzzo (IL 17)	Borroni (IL 23)	van Lumig (TNF)	Sator (TNF)	Ruiz Salas (IL 12/23)	García Gil (TNF)	García Gil (IL 12/23)
1st Line	14.1	33		80.9		69.2	70.2	3		65		
2nd Line	12.4	34	30	71.4	35.1	72.7	53.3	13	19	44	46.6	68.4
3rd/ 3rd+ Line				83.3		41.7	40			57		
4th/ 4th+ Line				20		44.4						

Study (Class of b/tsDMARD)

1st Line 2nd Line 3rd/ 3rd+ Line 4th/ 4th+ Line

Appendix S3: Graphs to show intra- and inter-study comparative PASI75/90/100 at 12-16 weeks in 1st to 4th line biologic and targeted small molecule treatment in included studies that recorded these outcome measures.

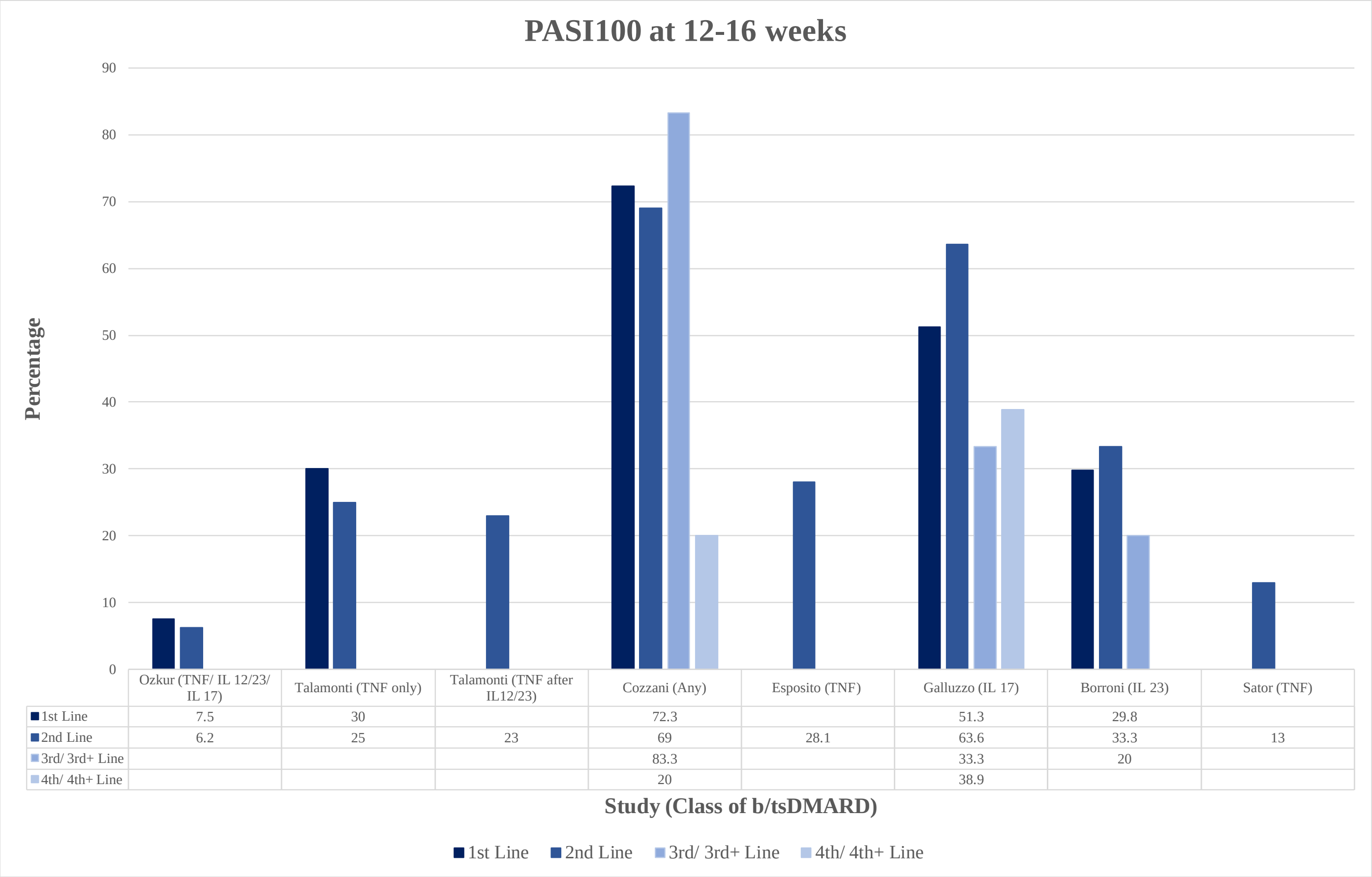

	Ozkur (TNF/ IL 12/23/ IL 17)	Talamonti (TNF only)	Talamonti (TNF after IL12/23)	Cozzani (Any)	Esposito (TNF)	Galluzzo (IL 17)	Borroni (IL 23)	Sator (TNF)
1st Line	7.5	30		72.3		51.3	29.8	
2nd Line	6.2	25	23	69	28.1	63.6	33.3	13
3rd/ 3rd+ Line				83.3		33.3	20	
4th/ 4th+ Line				20		38.9		
