# Supplementary material for: Effectiveness of sequential lines of biologic and targeted small molecule drugs in psoriasis: A systematic review and meta‐analysis
Source: Skin Health Dis. 2024 Feb 29;4(2):e350. doi: 10.1002/ski2.350 (PMC10988728; doi:10.1002/ski2.350)
Supplement: Supplementary file 4 — Table S1 [file SKI2-4-e350-s003.docx]

| Author, year, location | | Years of accrual | Study design and setting | Sample Size | Intervention b/tsDMARD | Class of intervention b/tsDMARD | Line of intervention b/tsDMARD | Prior biologic | Follow up (weeks) | Drop out | Outcome 1 | Result | Outcome 2 | Result | Outcome 3 | Result |
| --- | --- | --- | --- | --- | --- | --- | --- | --- | --- | --- | --- | --- | --- | --- | --- | --- |
| Papoutsaki  2007  Italy | | ND | Open label Prospective  Single centre | 19 | ada | TNFi | **3rd** | efa, eta or inf | 24 | 3 (16%) | PASI90 | 63% |  |  |  |  |
|  |  |  |  | 11 | ada | TNFi | **4th** | efa, eta or inf | 24 | 0 | PASI90 | 100% |  |  |  |  |
| Pitarch 2008  Spain | | ND | Observational  Prospective  Single centre | 8 | inf | TNFi | **1st** | None | 30 | 0 | PASI75 | 75% |  |  |  |  |
|  |  |  |  | 8 | eta | TNFi | **2nd** | inf | 12 | 0 | PASI75 | 63% |  |  |  |  |
| van Lumig  2010  Netherlands | | 2002-2010 | Observational  Prospective  Two centres | 30 | eta | TNFi | **1st** | None | 12 | 0 | PASI75 | 13% | PASI90 | 3% |  |  |
|  |  |  |  |  |  |  |  |  | 24 | 4 (13%) | PASI75 | 19% | PASI90 | 4% |  |  |
|  |  |  |  |  |  |  |  |  | 48 | 6 (20%) | PASI75 | 25% | PASI90 | 0% |  |  |
|  |  |  |  | 30 | ada |  | **2nd** | eta | 12 | 0 | PASI75 | 13% | PASI90 | 13% |  |  |
|  | |  |  |  |  |  |  |  | 24 | 2 (7%) | PASI75 | 7% | PASI90 | 0% |  |  |
|  | |  |  |  |  |  |  |  | 48 | 17 (57%) | PASI75 | 23% | PASI90 | 15% |  |  |
| Vender 2011  Canada | | 2009-2010 | Open label Prospective  Single centre | 10 | eta | TNFi | **2nd** | ada | 12 | 0 | Mean PASI reduction | 56% | PGA 0/1 | 70% |  |  |
|  |  |  |  |  |  |  |  |  | 24 |  | Mean PASI reduction | 68% | PGA 0/1 | 80% |  |  |
| Ruiz Salas 2012  Spain | | 2009-2011 | Observational  Retrospective  Single centre | 20 | ust | IL 12/23i | **1st** | None | 12 | 0 | PASI75 | 85% | PASI90 | 65% | Mean PASI | 1.7 |
|  |  |  |  |  |  |  |  |  | 24 |  | PASI75 | 85% | PASI90 | 75% | Mean PASI | 1.49 |
|  | |  |  |  |  |  |  |  | 36 |  | PASI75 | 95% | PASI90 | 80% | Mean PASI | 0.7 |
|  | |  |  | 9 | ust | IL 12/23i | **2nd** | ada, efa, eta, inf | 12 | 0 | PASI75 | 44% | PASI90 | 44% | Mean PASI | 4.4 |
|  | |  |  |  |  |  |  |  | 24 |  | PASI75 | 44% | PASI90 | 44% | Mean PASI | 5.4 |
|  | |  |  |  |  |  |  |  | 36 |  | PASI75 | 67% | PASI90 | 56% | Mean PASI | 4.3 |
|  | |  |  | 7 | ust | IL 12/23i | **3rd/ 3rd+** | ada, efa, eta, inf | 12 | 0 | PASI75 | 86% | PASI90 | 57% | Mean PASI | 3.5 |
|  | |  |  |  |  |  |  |  | 24 |  | PASI75 | 86% | PASI90 | 57% | Mean PASI | 7.9 |
|  | |  |  |  |  |  |  |  | 36 |  | PASI75 | 86% | PASI90 | 71% | Mean PASI | 1.4 |
| Piaserico 2014 Italy | | 2005-2010 | Observational  Prospective Psocare registry | 2933 | Ada, eta, inf | TNFi | **1st** | TNF | 16 | 0 | PASI75 | 30.6% |  |  |  |  |
|  |  |  |  |  |  |  |  |  | 24 |  | PASI75 | 42.5% |  |  |  |  |
|  |  |  |  |  |  |  |  |  | 52 |  | PASI75 | 67.5% |  |  |  |  |
|  |  |  |  | 105 | TNF all (see below for drug breakdown) | TNFi | **2nd** | TNF | 16 | 0 | PASI75 | 29% |  |  |  |  |
|  |  |  |  |  |  |  |  |  | 24 | 0 | PASI75 | 45.6% |  |  |  |  |
|  | |  |  |  |  |  |  |  | 52 | 0 | PASI75 | 74.1% |  |  |  |  |
|  | |  |  | 23 | eta | TNFi | **2nd** |  | 16 | 0 | PASI75 | 14.4% |  |  |  |  |
|  | |  |  |  |  |  |  |  | 24 | 0 | PASI75 | 29.8% |  |  |  |  |
|  | | |  | 39 | inf | TNFi | **2nd** |  | 16 | 0 | PASI75 | 26.6% |  |  |  |  |
|  | |  |  |  |  |  |  |  | 24 | 0 | PASI75 | 40.4% |  |  |  |  |
|  | |  |  | 43 | ada | TNFi | **2nd** |  | 16 | 0 | PASI75 | 38.3% |  |  |  |  |
|  | |  |  |  |  |  |  |  | 24 | 0 | PASI75 | 58% |  |  |  |  |
| Sator 2015  Austria | | 2008-2011 | Observational  Prospective  8 centres | 42 | ada | TNFi | **2nd** | efa, eta, inf | 12 |  | PASI75 | 52% | PASI90 | 19% | PASI100 | 13% |
|  |  |  |  |  |  |  |  |  | 24 |  | PASI75 | 71% | PASI90 | 52% | PASI100 | 16% |
|  | |  |  |  |  |  |  |  | 52 | 9 (21.4%) | PASI75 | 88% | PASI90 | 69% | PASI100 | 42% |
| Qiang 2016 Canada | | 2005-2014 | Observational  Retrospective  Multi-centre | 75 | ada, eta, inf | TNFi | **2nd** | ada, eta, inf, ust | 12 | ND | PASI75 or PGA 0/1 | 60% |  |  |  |  |
|  | |  |  | 43 | ust | IL 12/23i | **2nd** | ada, eta, inf, ust | 12 |  | PASI75 or PGA 0/1 | 84% |  |  |  |  |
|  | |  |  | 8 | ada, eta, inf | TNFi | **3rd** | ada, eta, inf, ust | 12 |  | PASI75 or PGA 0/1 | 50% |  |  |  |  |
|  | |  |  | 22 | ust | IL 12/23i | **3rd** | ada, eta, inf, ust | 12 |  | PASI75 or PGA 0/1 | 50% |  |  |  |  |
| Qiang cont | |  |  | 7 | ada, eta, inf | TNFi | **4th** | ada, eta, inf, ust | 12 |  | PASI75 or PGA 0/1 | 43% |  |  |  |  |
| Honda 2017  Japan | | 2010-2014 | Observational  Retrospective  Single centre | 275 | ada, inf, ust | TNFi/ IL 12/23i | **1st** | None | 14-16 | ND | PASI 75 | 59.1% | Mean PASI | 4.3 |  |  |
|  |  |  |  | 51 | ada, inf, ust | TNFi/ IL 12/23i | **2nd** | ada, inf, ust | 14-16 |  | PASI75 | 43.7% | Mean PASI | 2.9 |  |  |
| Reich 2017  US, Canada, Europe | | 2012-2016 | RCT sub-analysis | 83 | apr | PDE4i | **1st** | None | 16 | 6 (7%) | PASI75 | 39.8% |  |  |  |  |
|  |  |  |  |  |  |  |  |  | 52 | 27 (33%) | PASI75 | 53% |  |  |  |  |
|  |  |  |  | 83 | eta | TNFi | **1st** | None | 16 | 2 (2%) | PASI75 | 48.2% |  |  |  |  |
|  |  |  |  | 79 | apr | PDE4i | **2nd** | eta | 36 | 13 (16%) | PASI75 | 57% |  |  |  |  |
| Deza 2018  Spain | | ND | Observational Retrospective  7 centres | 20 | ixe | IL 17i | **1st** | None | 12 to 16 | ND | PASI75 | 100% |  |  |  |  |
|  |  |  |  | 19 | ixe | IL 17i | **2nd** | Any | 12 to 16 |  | PASI75 | 78.90% |  |  |  |  |
|  | |  |  | 21 | ixe | IL 17i | **3rd** | Any | 12 to 16 |  | PASI75 | 80.30% |  |  |  |  |
|  | |  |  | 40 | ixe | IL 17i | **≥ 4th+** | Any | 12 to 16 |  | PASI75 | 77.50% |  |  |  |  |
| Galluzzo  2018  Italy | | 2015-2017 | Observational  Retrospective  3 centres | 52 | sec | IL 17i | **1st** | Nil | 12 | ND | PASI75 | 76.9% | PASI90 | 69.2% | PASI100 | 51.3% |
|  |  |  |  |  |  |  |  |  | 24 |  | PASI75 | 78.1% | PASI90 | 75.0% | PASI100 | 59.4% |
|  |  |  |  |  |  |  |  |  | 52 |  | PASI75 | 95.7% | PASI90 | 91.3% | PASI100 | 87% |
|  | |  |  | 11 | sec | IL 17i | **2nd** | Any | 12 |  | PASI75 | 81.8% | PASI90 | 72.7% | PASI100 | 63.6% |
|  | |  |  |  |  |  |  |  | 24 |  | PASI75 | 81.8% | PASI90 | 81.8% | PASI100 | 81.8% |
|  | |  |  |  |  |  |  |  | 52 |  | PASI75 | 100% | PASI90 | 100% | PASI100 | 100% |
|  | |  |  | 18 | sec | IL 17i | **3rd** | Any | 12 |  | PASI75 | 66.7% | PASI90 | 41.7% | PASI100 | 33.3% |
|  | |  |  |  |  |  |  |  | 24 |  | PASI75 | 50% | PASI90 | 40% | PASI100 | 30% |
|  | |  |  |  |  |  |  |  | 52 |  | PASI75 | 80% | PASI90 | 60% | PASI100 | 60% |
|  | |  |  | 26 | sec | IL 17i | **4th+** | Any | 12 |  | PASI75 | 55.6% | PASI90 | 44.4% | PASI100 | 38.9% |
|  | |  |  |  |  |  |  |  | 24 |  | PASI75 | 56.3% | PASI90 | 43.8% | PASI100 | 31.3% |
|  |  | |  |  |  |  |  |  | 52 |  | PASI75 | 80% | PASI90 | 40% | PASI100 | 40% |
| Ganzetti 2018  Italy | | ND | Observational  Prospective  5 centres | 38 | ada, cer, gol, ust | TNFi/ IL 12/23i/ IL 17i | **2nd** | eta, ada, inf, ust | 8 | ND | PASI75 | 53% | Mean PASI | 7.8 |  |  |
|  |  |  |  |  |  |  |  |  | 16 |  | PASI75 | 89.40% | Mean PASI | 3.2 |  |  |
| Talamonti  2018  Italy | | 2010-2015 | Observational  Retrospective  5 centres | 154 | ada | TNFi | **1st** | Nil | 12 | 4 (3%) | PASI75 | 50% | PASI90 | 33% | PASI100 | 30% |
|  |  |  |  |  |  |  |  |  | 26 | 3 (2%) | PASI75 | 81% | PASI90 | 58% | PASI100 | 55% |
|  |  |  |  |  |  |  |  |  | 52 | 11 (7%) | PASI75 | 86% | PASI90 | 66% | PASI100 | 64% |
|  |  |  |  | 54 | ada | TNFi | **2nd** | TNF | 12 | 2 (4%) | PASI75 | 51% | PASI90 | 34% | PASI100 | 25% |
|  | |  |  |  |  |  |  |  | 26 | 2 (4%) | PASI75 | 83% | PASI90 | 52% | PASI100 | 44% |
|  | |  |  |  |  |  |  |  | 52 | 3 (6%) | PASI75 | 88% | PASI90 | 57% | PASI100 | 55% |
|  | | |  | 54 | ada | TNFi | **2nd** | ust | 12 | 1 (2%) | PASI75 | 43% | PASI90 | 30% | PASI100 | 23% |
|  | |  |  |  |  |  |  |  | 26 | 5 (9%) | PASI75 | 71% | PASI90 | 55% | PASI100 | 41% |
|  | |  |  |  |  |  |  |  | 52 | 9 (17%) | PASI75 | 82% | PASI90 | 64% | PASI100 | 49% |
| Esposito 2019, Italy | | 2010-2014 | Observational  Retrospective  4 centres | 115 | ada | TNFi | **2nd** | TNF | 12 | ND | PASI75 | 47.4% | PASI90 | 35.1% | PASI100 | 28.1% |
|  |  |  |  |  |  |  |  |  | 24 |  | PASI75 | 74.5% | PASI90 | 56.4% | PASI100 | 47.3% |
|  | | |  |  |  |  |  |  | 48 |  | PASI75 | 83.3% | PASI90 | 71.6% | PASI100 | 56.9% |
| Carpentieri 2020  France | | 2016-2018 | Observational  Retrospective  2 centres | 57 | sec | IL 17i | **1st** | None | 12 | ND | Mean PASI (+/-SD) | 5.3 ± 3.4 |  |  |  |  |
|  |  |  |  |  |  |  |  |  | 52 |  | Mean PASI (+/-SD) | 1.3 ± 2.5 |  |  |  |  |
|  | |  |  | 25 | sec | IL 17i | **2nd** | Any | 12 |  | Mean PASI (+/-SD) | 4.6 ± 3.4 |  |  |  |  |
|  | |  |  |  |  |  |  |  | 52 |  | Mean PASI (+/-SD) | 2.4 ± 3.3 |  |  |  |  |
|  | |  |  | 38 | sec | IL 17i | **3rd+** | Any | 12 |  | Mean PASI (+/-SD) | 3.8 ± 2.7 |  |  |  |  |
|  | |  |  |  |  |  |  |  | 52 |  | Mean PASI (+/-SD) | 3.7 ± 5.5 |  |  |  |  |
| Cozzani 2020  Italy | | 2006-2018 | Observational  Retrospective  Single centre | 88 | Any | Any | **1st** | None | 16 | ND | PASI75 | 95.7% | PASI90 | 80.9% | PASI100 | 72.3% |
|  |  |  |  | 63 | Any | Any | **2nd** | Any | 16 |  | PASI75 | 92.9% | PASI90 | 71.4% | PASI100 | 69% |
|  |  |  |  | 24 | Any | Any | **3rd** | Any | 16 |  | PASI75 | 94.4% | PASI90 | 83.3% | PASI100 | 83.3% |
|  |  |  |  | 20 | Any | Any | **4th+** | Any | 16 |  | PASI75 | 50% | PASI90 | 20.0% | PASI100 | 20% |
| Seneschal  2020  US/ Europe | | 2014-2017 | Observational  Prospective  Multi-centre | 507 | ada, cer, eta, inf, sec, ust | TNFi/ IL 12/23i/ IL 17i | **1st** | None | 26 | 149 (29%) | PASI100 | 25% |  |  |  |  |
|  |  |  |  |  |  |  |  | None | 52 | 195(38%) | PASI100 | 30% |  |  |  |  |
|  | |  |  | 203 | ada, cer, eta, inf, sec, ust | TNFi/ IL 12/23i/ IL 17i | **2nd** | Any | 26 | 56(28%) | PASI100 | 21% |  |  |  |  |
|  | |  |  |  |  |  |  | Any | 52 | 78(38%) | PASI100 | 22% |  |  |  |  |
|  | |  |  | 88 | ada, cer, eta, inf, sec, ust | TNFi/ IL 12/23i/ IL 17i | **3rd** | Any | 26 | 25(28%) | PASI100 | 19% |  |  |  |  |
|  | | |  |  |  |  |  | Any | 52 | 33(38%) | PASI100 | 18% |  |  |  |  |
|  | |  |  | 48 | ada, cer, eta, inf, sec, ust | TNFi/ IL 12/23i/ IL 17i | **4th** | Any | 26 | 13(27%) | PASI100 | 14% |  |  |  |  |
|  | |  |  |  |  |  |  | Any | 52 | 18(38%) | PASI100 | 10% |  |  |  |  |
| Borroni 2021  Italy | | ND | Observational  Retrospective  Multi-centre | 47 | ris | IL 23i | **1st** | None | 16 | 0 | PASI75 | 89.4% | PASI90 | 70.2% | PASI100 | 29.8% |
|  |  |  |  |  |  |  |  |  | 40 | 0 | PASI75 | 100% | PASI90 | 93.6% | PASI100 | 70.2% |
|  |  |  |  | 15 | ris | IL 23i | **2nd** | Any | 16 | 0 | PASI75 | 93% | PASI90 | 53.3% | PASI100 | 33.3% |
|  |  |  |  |  |  |  |  |  | 40 | 0 | PASI75 | 93.3% | PASI90 | 93.3% | PASI100 | 66.7% |
|  | |  |  | 15 | ris | IL 23i | **3rd+** | Any | 16 | 0 | PASI75 | 66.7% | PASI90 | 40% | PASI100 | 20% |
|  | |  |  |  |  |  |  |  | 40 | 0 | PASI75 | 100% | PASI90 | 53.3% | PASI100 | 33.3% |
| Ozkur 2021  Turkey | | 2007-2019 | Observational  Retrospective  6 centres | 427 | ada, eta, inf, sec, ust | TNFi/ IL 12/23i/ IL 17i | **1st** | None | 12 | 0 | PASI75 | 25.7% | PASI90 | 14.1% | PASI100 | 7.5% |
|  |  |  |  | 145 | ada, eta, inf, sec, ust | TNFi/ IL 12/23i/ IL 17i | **2nd** | ada, eta, inf, sec, ust | 12 | 0 | PASI75 | 22.7% | PASI90 | 12.4% | PASI100 | 6.2% |

Abbreviations: *ada* Adalimumab, *apr* Apremilast, *cer* Certolizumab pegol, *efa* Efalizumab, *eta* Etanercept, *gol* Golimumab, *inf* Infliximab, *ixe* Ixekizumab, *ris* Risankizumab, *sec* Secukinumab, *ust* Ustekinumab, *TNFi* Tumour necrosis factor- alpha inhibitor, *ILxi* Interleukin x inhibitor, *PDE4i* Phosphodiesterase 4 inhibitor, *ND* Not documented, *PASI* Psoriasis Area and Severity Index, *PGA* Physician Global Assessment, *SD* Standard Deviation, *US* United States
