# Supplementary material for: Effectiveness of sequential lines of biologic and targeted small molecule drugs in psoriasis: A systematic review and meta‐analysis
Source: Skin Health Dis. 2024 Feb 29;4(2):e350. doi: 10.1002/ski2.350 (PMC10988728; doi:10.1002/ski2.350)
Supplement: Supplementary file 5 — Table S2 [file SKI2-4-e350-s005.docx]

| **Author, year, country** | **Study design and setting** | **Years of accrual** | **Sample Size** | **Rate of drop out** | **Prior b/ts** | **Intervention b/ts** | **Class of intervention b/ts** | **Line of intervention b/ts** | **Follow up (months)** | **PsO Outcome** | **Result** |
| --- | --- | --- | --- | --- | --- | --- | --- | --- | --- | --- | --- |
| **García Gil, 2018, Spain** | Observational  Retrospective  Single centre | 2011-2015 | 15 | 0 | Etanercept | Adalimumab | TNFi | 2nd | 3 | PASI 90 | 46.6% |
|  |  |  | 19 | 0 | Etanercept | Ustekinumab | IL12/23i | 2nd | 3 | PASI 90 | 68.4% |

Abbreviations: *b/ts* biologic or targeted small molecule drug *TNFi* Tumour necrosis factor- alpha inhibitor, *IL 12/23i* Interleukin 12/23 inhibitor, *PASI* Psoriasis Area and Severity Index
